# Preliminary Evidence of the Role of Medial Prefrontal Cortex in Self-Enhancement: A Transcranial Magnetic Stimulation Study

**DOI:** 10.3390/brainsci10080535

**Published:** 2020-08-08

**Authors:** Birgitta Taylor-Lillquist, Vivek Kanpa, Maya Crawford, Mehdi El Filali, Julia Oakes, Alex Jonasz, Amanda Disney, Julian Paul Keenan

**Affiliations:** 1 Department of Biology, University of Virginia, Charlottesville, VA 22904, USA; birgittatl@gmail.com; 2 Department of Engineering, Northeastern University, Boston, MA 02115, USA; vkanpa184@gmail.com; 3 Department of Biology, Northeastern University, Boston, MA 02115, USA; mayatcrawford@gmail.com; 4 Department of Psychology, Wesleyan University, Middletown, CT 06457, USA; melfilali2001@gmail.com; 5 Department of Psychology, Hamilton College, Clinton, NY 13323, USA; joakes@hamilton.edu; 6 Department of Biology, George Washington University, Washington, DC 20052, USA; Amjonasz@gmail.com; 7 Department of Dentistry, University of Indiana, Bloomington, IN 47405, USA; akdisney2@gmail.com; 8 Cognitive Neuroimaging Laboratory, 207 Science Hall, Montclair State University, Upper Montclair, NJ 07043, USA

**Keywords:** social pressure, deception, medial prefrontal cortex, overclaiming, self-deception, transcranial magnetic stimulation, self-enhancement, social monitoring

## Abstract

Humans employ a number of strategies to improve their position in their given social hierarchy. Overclaiming involves presenting oneself as having more knowledge than one actually possesses, and it is typically invoked to increase one’s social standing. If increased expectations to possess knowledge is a perceived social pressure, such expectations should increase bouts of overclaiming. As the medial prefrontal cortex (MPFC) is sensitive to social pressure and disruption of the MPFC leads to decreases in overclaiming, we predicted that transcranial magnetic stimulation (TMS) applied to the MPFC would reduce overclaiming and the effects would be enhanced in the presence of social pressure. Twelve participants were given a test in which half of the words were real and half were fake, and they were asked how well they knew each word. They were not told that any of the words were fake. Half of the participants were exposed to social pressure while the other half were not. Following TMS delivered to the MPFC, overclaiming rates decreased, specifically under conditions of high social pressure. Medial PFC TMS did not influence real word responses and real words did not interact with the MPFC and social pressure. These preliminary findings support the significant role the MPFC plays in social cognition and the importance of the MPFC in mediating socially meaningful situations. We suggest the role of the MPFC as being highly influenced by the premium placed on social manipulation in human evolution.

## 1. Introduction

There are numerous factors that contribute to success within a social interaction. Often examined in social neuroscience is the influence of ego management and self-presentation [1,2]. An individual’s ability to present oneself in a positive light has significant evolutionary benefits including, but not limited to, reproductive potential and threat management [3]. Self-presentation is often steeped in deception, a critical component of social communication in humans. Snyder [4] in fact described monitoring of the self as the process through which people regulate their own behavior in order to be perceived in a favorable way and implicated that self-awareness had a primary basis in self-deception [5].

Overclaiming is the term often ascribed to individuals claiming to have knowledge when they in fact do not [6]. This pretense of knowledge is thought to be one of many attributes that make up self-enhancement which involves falsely increasing one’s statues [7]. The typical method used for testing overclaiming is to present words that are not actual words, but adhere to typical grammatical rules (e.g., ‘triannic’, which could be a word). When participants view these words along with actual words, they often claim that they know them when in fact the words are fake and by definition, unknowable. This method has been widely used [8], though there is evidence that overclaiming measured this way may be influenced by other variables [9]. Typical deviations from Palhaus’s original design almost always seek to increase overclaiming and include tapping into the participants’ areas of expertise [10].

We are far from a full understanding of overclaiming. There are likely numerous fundamental influences at play such as a memory bias in which humans often overreport familiarity with stimuli in general and therefore may be biased to think words are known rather than not known [11]. A self-confirmation bias in terms of false memories may also be at play [12]. This is particularly true when there is no consequence for falsely reporting knowledge [2]. It is not even known the degree to which the phenomenon is implicit or explicit [8] as is the case with most of deception [13].

Despite the ambiguities, it is generally agreed upon, that at some level, respondents are not willing to admit ignorance and this resistance to admit lack of knowledge stems from a desire to present oneself favorably [14,15]. Specifically, Bradley [15] highlights that this overconfidence is especially likely to occur in areas of perceived expertise which may be tapping into Trivers original notion that self-deception encourages over-confidence even in the light of contradictory evidence [16,17]. Overall, most of the research on overclaiming has focused on the degree to which it represents ego-protecting responses (such as those seen in narcissism) that are linked to overclaiming [18].

The medial prefrontal cortex (MPFC) is a focal point of both social awareness (i.e., being aware that other individuals are monitoring oneself) and overclaiming. The MPFC is responsible for ‘social navigation’ and knowing and applying social ‘rules’ [19]. Moreover, while adult cases are often highlighted, the MPFC is critical during childhood and adolescent development in terms of social responding [20,21,22].

Social monitoring and social responding as well as self-awareness in the context of social stimuli [23,24] involve MPFC circuits, though clearly other regions such as the amygdala play a role [25]. Furthermore, patients with frontal lobe damage often present with judgment errors including overestimating [26]. Functional neuroimaging studies have revealed that biased self-estimation is correlated with MPFC and ventral prefrontal cortex (VPFC) activity [27].

There is evidence for a causal link between the MPFC and overclaiming. Specifically, when transcranial magnetic stimulation (TMS) was delivered to the MPFC, bouts of overclaiming were significantly reduced [5], which has also been observed with tDCS (transcranial direct current stimulation [28]). Specifically, by temporarily inhibiting the MPFC with single-pulse TMS, rates of overclaiming went down significantly compared to Sham TMS. However, social pressure was not varied, and it is not known how increased (or decreased) social pressure would influence the MPFC’s role in overclaiming. Recent evidence has emerged that faking both good (i.e., enhancing a person’s status in a positive direction) and bad (i.e., intentionally exaggerating to make oneself appear more negative) as well as responding in a socially desirable manner, are mediated via the MPFC. Furthermore, it was reported that faking good may in fact be the default mode of the MPFC which suggests that disruption of this region should have the most dramatic impact under conditions when one should be under the most pressure to deceive in a favorable direction [29]. It is unknown as of now if this relationship is causal.

Because social pressure and overclaiming appear to be mediated at least in part by networks within the MPFC, it is reasonable to assume that MPFC disruption would have a significant influence on overclaiming when a person feels pressured to self-enhance. Specifically, we predicted that under conditions of social pressure, TMS delivered to the MPFC would have a greater impact on overclaiming than in conditions of no social pressure. Thus, we predict a greater disruption to overclaiming when TMS is applied to the MPFC during the increased social pressure condition compared to no social pressure condition.

## 2. Methods

### 2.1. Participants

Twelve university students were recruited (6 males and 6 females, age 18–65; M = 20.4; SE = 1.1) via flyer and word of mouth for the study. All participants were paid 25 dollars for their participation and were treated in accordance with guidelines set forth by the Internal Review Board at Montclair State University and guidelines of the American Psychological Association. All TMS was delivered within the parameters provided by Wasserman [30]. As per local IRB guidelines, all TMS was delivered by the PI (approved by Montclair State University IRB: IRB-16-17-424, TMS and the Self).

### 2.2. Materials

A Magstim, single-pulse TMS device was used for all stimulation. A 70-mm figure-of-eight coil was used throughout the experiment. All stimuli were presented on a Dell laptop computer with a 17” display using proprietary in-house software. All triggering occurred through in-house modified Arduino circuit boards that time-locked TMS with stimuli presentation (see below).

### 2.3. Stimuli

All items were drawn from the comprehensive list adopted by Paulhus et al. [6] and further modified. The items contained words referring to historical names, events, books, fine arts, poems, literature, authors, social science, physical science, law and popular culture. The foil words were created to appear as if they legitimately belonged to one of these categories. As in Paulhus et al. [6] the words were created to appear to be plausible members of the English language.

All participants were students enrolled at Montclair State University. Participants were randomly placed into either the social pressure group or the no social pressure group (however, both groups were equivalent in terms of sex). All participants were treated identically between the groups other than the instructions they received concerning the test. The instructions were as follows:

**No social pressure group instructions:** You are going to be tested on a series of words. These words are drawn from an advanced vocabulary list. The words have been rated extremely difficult and all of the participants we have tested thus far have had a difficult time with this test. Therefore, it is normal to not know many of these words. In fact, we recently ran this study with Princeton University students and even those students did poorly. If you know the word press the ‘/’ key. If you do not know the word, press the ‘Z’ key. Speed is important, but so is accuracy. Please note, we will not ask you what the word means. We are trusting you-if you say you know the word, we will believe you. Likewise, if you say you do not know the word we will believe that as well. Again, be as accurate and as fast as possible and remember, there is no pressure to do well. Do you have any questions?

**Social pressure group instructions:** You are going to be tested on a series of words. These words are drawn from a basic, simple vocabulary list. The words have been rated extremely easy and all of the participants we have tested thus far have had an easy time with this test. Therefore, you should know most of these words. In fact, we recently ran this study with High School students and even those students did well. If you know the word press the ‘/’ key. If you do not know the word, press the ‘Z’ key. Speed is important, but so is accuracy. Please note, we will not ask you what the word means. We are trusting you-if you say you know the word, we will believe you. Likewise, if you say you do not know the word we will believe that as well. Again, be as accurate and as fast as possible. Please, do your best and remember that this list is pretty easy. Do you have any questions?

These instructions were given following the Motor Threshold determination and prior to the main experiment. Questions received were of a general nature and testing then began.

### 2.4. Transcranial Magnetic Stimulation (TMS) Procedure

Wasserman’s [30] guidelines were used to set the limits of stimulation throughout the testing sessions. The testing was executed in two phases: motor threshold determination and the experiment proper. Participants were initially fitted with a tight Lycra swim cap. Suprathreshold TMS pulses were delivered to locate the region that provided the greatest MEP response to the contralateral Abductor Pollicis Brevis (APB) muscle. The coil was relocated across the scalp until the most responsive region was found that induced MEPs of maximal peak-to-peak amplitude. Determination of individual MT was employed using procedures outlined by IFCN (International Federation of Clinical Neurophysiology [31]), such that threshold was established when 50% (5 of 10) of the TMS pulses delivered induced a measured MEP of >50 µV. All active stimulation was delivered at 90% MT during the experiment proper. All MT measurements were made via BioPack MP150 amplifiers and software. Once the MT intensity was determined, the cap was marked in the 10/20 International system for EEG electrode positions.

The regions of interest were the precuneus (Pz), the MPFC and the SMA. Cortical placement was identical to those used in similar studies [2,10]. In these previous studies, only the MPFC significantly impacted over-claiming and self-enhancement. To determine the locations, one third of the distance, nasion to inion, was measured for each participant. MPFC was 1.5 cm anterior to this location, and SMA was identified as being 3 cm posterior to this location. The coil was oriented parallel to the mid-sagittal line for all stimulation with the handle pointed in a posterior orientation (except for APB MT determination in which the coil was held at ~45° from the hemispheric line). The depth of cortical stimulation is no greater than 2 cm, ensuring that the initial effects of TMS are concentrated to the areas of interest [30].

Baseline performance was measured by a Sham condition in which TMS stimulation was delivered through the coil, but not to the participants’ head. During sham, the TMS coil was held at 90° orientation and held over Cz (standard 10/20 system coordinates). Because the regions (MPFC and SMA) are somewhat adjacent, single-pulse TMS was employed to avoid cortical spread. The coil was held manually [32] to ensure quick shifting of blocks as they changed approximately once per minute. For all testing sessions, participants wore protective earplugs to prevent transient threshold shifts caused by the acoustic artifact generated by the discharge of the TMS coil [30].

### 2.5. Measures of Overclaiming

The methods employed were those similar to Amati et al. [5]. The list of words (both real and fake) was divided into 4 blocks containing 36 words per block (Table A1). For each block 50% of the words were real terms (e.g., monochrome, zygote), and 50% were fake terms (e.g., fibia, triannic). Therefore, TMS to each of the four brain regions was delivered during 36 word presentations. All words were randomized, and all lists were counterbalanced across participants. The order of all brain region sites was randomized (Appendix A). All words within a block were also randomly presented.

Participants indicated their response (yes or no) via a standard keyboard. Trials began once the participant demonstrated comfortability with the keyboard and layout; practice trials were not given. For all trials, TMS was delivered 300 ms after the word appeared on the screen. Response times (RT) were measured as the amount of time after the TMS pulse.

## 3. Results

The overall reaction time was 406.07 ms (SE = 7.06). The average reaction time for ‘yes’ responses (indicating the person knew the word) was 395.59 (SE = 10.87) and for no responses it was 424.67 (SE = 6.96). This difference was found to be statistically insignificant (*t*(11) = 1.43, *p* = 0.18; Bayes Factor = 1.9; Null vs. Alternative). In terms of overall response types, a response of ‘yes’ was indicated 63.97% of the time and a response of ‘no’ was indicated 36.03% of the time. Because half of the words were real, a ‘perfect’ result would be 50/50. To test if there was a response bias, a chi-squared of all the trials was conducted. It was found that there was a significant bias (X^2^(1) = 103.40, *p* < 0.0001). This indicated that participants felt compelled to respond ‘yes’ to knowing words.

Following up on this, we sought to determine if overclaiming occurred. The overall percentage of ‘yes’ responses to real words was 64.25% (SD = 4.59%) and the overall percentage of ‘yes’ responses to fake words was 63.76% (SD = 3.76%). There was no difference between the word lists (real and fake) in terms of reported knowledge (X^2^(1) = 0.02, *p* > 0.05).

To test for reaction time differences, a 2 × 4 × 2 (response × brain region × word type) repeated measures ANOVA was performed. It was found that there was no overall three-way interaction (F (3,33) = 0.55, *p* > 0.05; BF_null_ = 3.5). All two-way interactions were also statistically insignificant: response x brain region (F(3,33) = 0.336, *p* > 0.05; BF_null_ = 3.9), response x word type (F(1,11) = 0.09, *p* > 0.05; BF_null_ = 4.4) and brain region x word type (F(3,33) = 0.18, *p* > 0.05; BF_null_ = 4.2). There was no significant main effect for response (F(1,11) = 1.52, *p* > 0.05; BF_null_ = 2.3) or word type (F(1,11) = 0.33, *p* > 0.05; BF_null_ = 3.9). There was a significant main effect for brain region (F(3,33) = 3.19, *p* < 0.04; BF_null_ = 1.2). Post hoc LSD tests revealed that the reaction time during MPFC TMS was significantly longer (M = 443.12, SE = 9.53) than sham (M = 386.63, SE = 15.78, *p* > 0.05) and PZ (M = 395.91, SE = 9.53, *p* > 0.04). This result indicated that responses were delayed when the MPFC was disrupted.

In terms of responses, a similar analysis was performed. Since no/yes responses are directly related, only yes responses were analyzed in a 2 × 4 repeated measures ANOVA (word type x brain region). It was found that there was no interaction (F(3,33) = 0.28, *p* > 0.05; BF_null_ = 4.0). There was also no main effect for word type (F(1,11) = 1.87, *p* > 0.05; BF_null_ = 1.9). There was a main effect for brain region (F(3,33) = 6.04, *p* > 0.002; BF_alternative_ = 2.1). The only differences were found when Sham (M = 10.0, SE = 0.33) was compared to all other groups: MPFC (M = 7.88, SE = 0.42, *p* < 0.007), PZ (M = 8.80, SE = 0.30, *p* < 0.005) and SMA (M = 8.63, SE = 0.32, *p* < 0.02).

**Figure 1:**
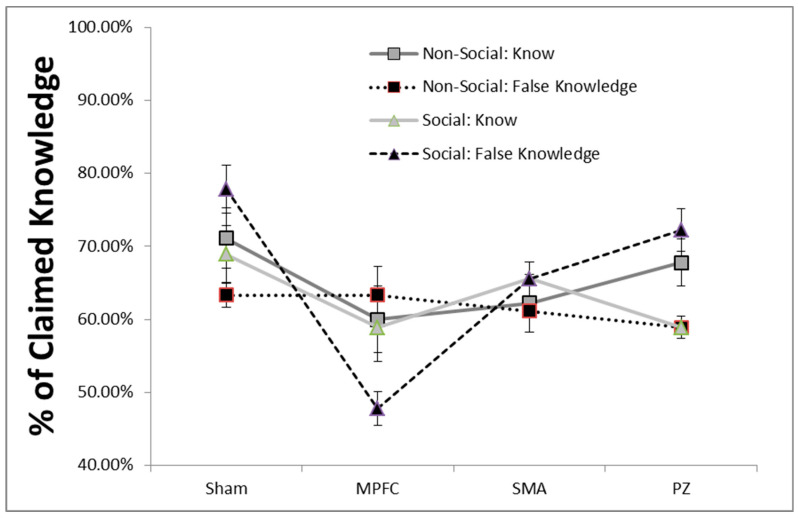
Transcranial magnetic stimulation (TMS) delivered to the medial prefrontal cortex (MPFC) had a significant impact in terms of percent of claimed knowledge. The vertical axis indicates the percent of yes responses for either real words (knowledge) or fake words (false knowledge). It was found that TMS delivered to the MPFC significantly reduced the participants’ overclaiming (*p* < 0.04).

To test the main hypotheses surrounding social pressure, a 2 × 2 × 4 (social pressure × word type × brain region) mixed ANOVA was run in terms of the number of ‘yes’ responses. A significant three-way interaction was found (F(1,30) = 3.11, *p* < 0.04; BF_null_ = 1.2). To determine the precise nature of this interaction, we examined real and fake words independently. It was found that there was no interaction (social pressure x brain region) for real words (F(3,30) = 0.72, *p* > 0.05; BF_null_ = 3.2). However, for fake words, there was a significant social pressure x brain region interaction (F(3,30) = 4.77, *p* < 0.008 BF_alternative_ = 3.8). This suggested that social pressure interacted uniquely with brain areas for overclaiming. In the Sham condition, when social pressure was applied, people tended to ‘know’ fake words more (*t*(11) = 2.74, *p* < 0.02; BF_alternative_ = 3.1). This demonstrated a baseline influence of social pressure. TMS delivered to PZ resulted in a similar significant difference between the social (M = 11.0, SE = 0.68) and non-social groups (M = 9.0, SE = 0.26, *t*(11) = 2.61, *p* < 0.03; BF_alternative_ = 2.6). That difference disappeared when the SMA was disrupted (*t*(11) = −0.18, *p* > 0.05 BF_null_ = 4.5). Transcranial Magnetic Stimulation did the opposite when applied to the MPFC: The participants were less likely to overclaim in the social condition (M = 6.33, SE = 0.62) when compared to the non-social condition (M = 8.50, SE = 0.67, *t*(11) = 2.38, *p* < 0.04; BF_alternative_ = 1.9; Figure 1).

We also analyzed these data in terms of reaction time. Employing a 2 × 2 × 2 × 4 (social pressure × word type × response × brain region) mixed ANOVA, it was found that there were no significant differences (all *p*’s > 0.05). The only trend observed across all interactions and main effects was the social pressure × response interaction (*p* = 0.06). For the non-social pressure group, there was no difference between the ‘no’ response (M = 395.61, SE = 20.13) and the ‘yes’ response (M = 407.60, SE = 12.41; (*t*(5) = −0.41, *p* > 0.05: BF_null_ = 4.2). There was a difference between ‘no’ response (M = 442.61, SE = 9.80) and the ‘yes’ response (M = 381.19, SE = 12.55; *t*(5) = 3.25, *p* < 0.02; BF_alternative_ = 6.6) for the social pressure group. This interaction indicated that when pressured socially, it took additional time to respond ‘no’. Caution is urged in any interpretation of this finding as the omnibus test was only a trend.

## 4. Discussion

It was found that when participants were exposed to social pressure they increased their rate of ‘knowing’ fake words compared to when no social pressure was applied. This finding emerged despite the finding that fake words were reported to be known at about the same rate as real words. Additionally, when social pressure was applied, there was a trend to take longer to reject false words. Taken together, there appears to be a default state of responding ‘yes’ to knowing words (real or fake) and that social pressure increases the desire to respond in a socially beneficial manner. In terms of TMS, disrupting the MPFC when paired with social pressure lowered knowing fake words, indicating an increase in honest responding. Based on the current preliminary evidence, we conclude that it is likely that the MPFC has a causal role in overclaiming and that applying social pressure increases the importance of the MPFC.

We have previously found using a similar paradigm that the response rate of ‘knowing’ words is not different between fake and real words, which results in a significant amount of false positives [5]. Similar to Palhaus’s findings [33,34], we conclude that faking knowledge is socially mediated and that the risk of being seen not knowing something is greater than claiming to know something that you do not. This indicates humans may bias towards self-deception as previously reported [16,35,36] and it may relate to other findings such as those indicating it takes much evidence to sway an individual from a false belief [37].

The MPFC plays a critical role in testing reality [19,38], self-knowledge [39,40] and more specifically for this study, falsifying one’s reported knowledge [5], abilities and traits [29] as well as monitoring social reactions of others in regards to the self [41].It is therefore not surprising to observe disruption of the MPFC has an influence on false responding. Because TMS had an influence on fake words, but not real words, we speculate that disruption of the MPFC is not uniform in its cognitive influence. It is not known why such a difference exists proximately as it could be any number of different factors (e.g., cognitive load, unfamiliarity, anxiety, etc.). However, as a preliminary finding, we assume that these data suggest that false reporting involves unique structures of the brain though this is highly speculative [38].

Other social pressures need to be applied to test the generalizability of our findings as do participant expectations [42]. For example, our instructions invoked a social pressure given by one individual (i.e., the experimenter) which may differ from group pressure or pressure given by an intimate other. While significant evidence exists that the MPFC would respond similarly, we do not know that to be the case. While overclaiming was chosen as a measure of self-enhancement, we also do not know if all cases of self-enhancement (e.g., over-evaluating one’s driving ability) would be influenced similarly. Further testing needs to be done before it is known if all cases of social pressure influence all cases of self-enhancement. While we do not know the outcomes, we will speculate that social pressures with more serious evolutionary outcomes than faking word knowledge (e.g., pressure with direct reproductive consequences) would be even more influenced by MPFC disruption.

### Caveats

We consider these data preliminary as the sample size was low (*n* = 6 per group). These data should be replicated in a larger sample. We also consider these data preliminary due to an overall lack of literature concerning the role overclaiming has in neuroscience and cognition in general [43]. Not being able to answer if the individual truly believes their response (or if they are ignorant that they are falsely responding) is problematic. While this harkens to the debates of the definition of the term ‘self-deception’ itself and remains beyond the scope of this study, it is important to acknowledge that overclaiming as a dependent variable still needs investigation from a purely psychological perspective. We believe that neuroimaging may be of some help in detangling if participants are aware of their false claiming. We further consider these data preliminary as the sample size was low. These data should be replicated in a larger sample.

In conclusion, we report that the MPFC plays a large role in navigating the social world. Social pressure was found to influence false knowledge such that TMS stimulation applied to MPFC resulted in less over-claiming. These data indicated that the MPFC is involved in the interpretation of social pressure and suggestive of the importance the MPFC may play in evaluating social pressure to ‘be smart’ or ‘know it all’. While preliminary, these data are consistent with other research detailing overclaiming being tied to the MPFC.

## Appendix A

Order of random brain site stimulation by participant (#): a = Sham; b = MPFC, c = SMA, d = PZ

1acdb, 2bdca, 3bacd, 4dcab, 5adcb, 6dabc, 7bdac, 8cbad, 9bdca, 10cadb, 11abdc, 12dbca

**Table A1:** List of words used in the experiment.

Real Words	Fake Words
Lepidoptery	Midfloral
Follicle	Vorax
Burette	Midternal
Lobotomy	Perchery
Cognizant	Dex
Entomology	Inosmosis
Allele	Tribution
Amorphous	Prevoid
Polymerization	Suffaltible
Scalar	Halible
Zygote	Formatic
Viscosity	Eratifinatic
Quark	Ellictitive
Artery	Myric
Dogmatic	Nampercilious
Exocytosis	Precilious
Morass	Exultion
Opulent	Tibula
Tribulation	Saprocy
Insipid	Sepititious
Supercilious	Oblongular
Laud	Rancidious
Xenophobia	Geolithic
Omnipotent	Trisectomy
Unequivocal	Recticious
Abdicate	Gruffle
Flagellum	Plumistic
Extravagant	Berythium
Iridescent	Allurium
Disgruntled	Florabane
Colloquial	Eloquacious
Perpetuity	Oblivia
Zygomaticus	Prevoid
Foment	Catosynthesis
Rhizome	Midternal
Nazca lines	Bellignorant
Intrinsic	Promedic
Homo erectus	Bluther
Unscrupulous	Lchistamine
Soliloquy	Reccentric
Justification	Geomorphile
The Defenestration of Prague	Paradign
Indefinite	Accentrax
Clairvoyant	Paradocial
Auspicious	Prerecuite
Trivial	Morphinil
Maleficent	Yusteric
Fission	Protrake
Inclement	Gauderate
Aglet	Quartom
Umlaut	Polycerics
Latus rectum	Dright
Paraben	Hapsate
Palm frond	Carcolepsy
Turpentine	Jentacular
Philtrum	Ternal
Uvula	Mimsy
Cornichon	Gleek
Directrix	Glumium
Occipital	Skoch
Duodenum	Flubium
Clad	Bleeale
Dwell	Tongressiant
Impel	Lymispre
Accede	Guniquated
Rebuke	Agnosary
Cede	Esagnacious
Facile	Nuwmerts
Entreat	Craderdon
Earnest	Invinceclace
Exalt	Logistorgian
Ensue	Greepaling
